# Vascular smooth muscle cells in response to cholesterol crystals modulates inflammatory cytokines release and promotes neutrophil extracellular trap formation

**DOI:** 10.1186/s10020-024-00809-8

**Published:** 2024-03-22

**Authors:** Jishamol Thazhathveettil, Ashok Kumar Kumawat, Isak Demirel, Allan Sirsjö, Geena Varghese Paramel

**Affiliations:** 1 Cardiovascular Research Centre, School of Medical Sciences, Faculty of Medicine and Health, Örebro University, 70182 Örebro, Sweden; 2 School of Medical Sciences, Örebro University, 70182 Örebro, Sweden

**Keywords:** Cholesterol crystal, Vascular smooth muscle cells, Atherosclerosis, Neutrophil extracellular traps, Inflammation, Cardiovascular disease, Neutrophils, Interleukin-33 (IL-33)

## Abstract

**Background:**

The formation and accumulation of cholesterol crystals (CC) at the lesion site is a hallmark of atherosclerosis. Although studies have shown the importance of vascular smooth muscle cells (VSMCs) in the disease atherosclerosis, little is known about the molecular mechanism behind the uptake of CC in VSMCs and their role in modulating immune response.

**Methods:**

Human aortic smooth muscle cells were cultured and treated with CC. CC uptake and CC mediated signaling pathway and protein induction were studied using flow cytometry, confocal microscopy, western blot and Olink proteomics. Conditioned medium from CC treated VSMCs was used to study neutrophil adhesion, ROS production and phagocytosis. Neutrophil extracellular traps (NETs) formations were visualized using confocal microscopy.

**Results:**

VSMCs and macrophages were found around CC clefts in human carotid plaques. CC uptake in VSMCs are largely through micropinocytosis and phagocytosis via PI3K–AkT dependent pathway. The uptake of CC in VSMCs induce the release inflammatory proteins, including IL-33, an alarming cytokine. Conditioned medium from CC treated VSMCs can induce neutrophil adhesion, neutrophil reactive oxygen species (ROS) and neutrophil extracellular traps (NETs) formation. IL-33 neutralization in conditioned medium from CC treated VSMCs inhibited neutrophil ROS production and NETs formation.

**Conclusion:**

We demonstrate that VSMCs due to its vicinity to CC clefts in human atherosclerotic lesion can modulate local immune response and we further reveal that the interaction between CC and VSMCs impart an inflammatory milieu in the atherosclerotic microenvironment by promoting IL-33 dependent neutrophil influx and NETs formation.

**Supplementary Information:**

The online version contains supplementary material available at 10.1186/s10020-024-00809-8.

## Introduction

Atherosclerosis is a chronic inflammatory disease characterized by a dysfunctional interplay between immune response and lipids. The progression of atherosclerotic plaque involves interaction between vascular smooth muscle cells (VSMCs), endothelial cells and immune cells, leading to recruitment of neutrophils, monocytes, lymphocytes, mast cells and release of inflammatory proteins (Libby 2002).

Cholesterol crystals (CC), a potential biomarker of atherosclerosis is the most abundant crystalline structure found in the atherosclerotic plaque (Abela et al. 2009). Since its discovery, CC have been observed in many disease conditions, including kidney diseases, gallstone formation, periodontitis, myocardial infarction, ocular diseases, abdominal aortic aneurism, and even central nervous anomalies (Sedaghat and Grundy 1980; Scolari et al. 2000; Li et al. 2017; Chen and Popko 2018). In human atherosclerotic plaque, CC formation is influenced by many physiochemical factors including pH and temperature of the milieu, presence of excess of calcium, saturation of free cholesterol, extent of hydration of free cholesterol molecules (Nidorf et al. 2020). Lethality of CC has been proven by many studies. Needle shaped and plate shaped CC which expands in volume and pierces the blood vessel can cause rupture of the plaque with its sharp edges (Abela 2010a, b; Lim et al. 2011). Moreover, plaque rupture can facilitate breaching of CC and formation of CC emboli, leading to obstruction of medium to small arteries (Pervaiz et al. 2018). Furthermore, CC emboli are known to induces tissue inflammation and distal ischemia (Pervaiz et al. 2018) indicating that CC plays a lethal role in the later stages of atherosclerosis (Abela et al. 2009). CC are present in all stages of atherogenesis and its appearance in the atherosclerotic lesions coincides with the first appearance of inflammatory cells (Duewell et al. 2010). CC represents an endogenous danger signal that induce plaque inflammation via NLRP3 mediated IL-1β production during atherogenesis (Abela et al. 2009; Abela 2010a, b; Duewell et al. 2010; Varghese et al. 2016). The formation and accumulation of CC are known to promote inflammation by activation of NLRP3 inflammasome and release of potent pro-inflammatory cytokine, IL-1β in macrophages causing cellular dysfunction and acute vascular injury (Duewell et al. 2010; Lopez-Castejon and Brough 2011). In vascular endothelium, CC increase vascular permeability by disrupting adherens junctions and promote interaction between endothelial cells and monocytes (Mani et al. 2018; Pichavaram et al. 2019). Studies from Benoit group have demonstrated that CC formation is initiated in the tight association with the death of intralesional SMCs during the transition of fatty streak to fibroatheroma of human atherosclerotic plaque. Human VSMCs loaded with cholesterol can produce CC by inducing collagen dependent changes in cholesterol metabolism and autophagy flux (Ho-Tin-Noé et al. 2017).

CC are known to act as endogenous danger signal to induce sterile inflammatory immune response in atherosclerosis. IL-33, an IL‐1‐related cytokine is known to augment sterile inflammation in cardiovascular diseases including atherosclerosis (Liew et al. 2016; Sun et al. 2021). An elevated concentration of serum IL-33 and its receptors were reported in high-risk carotid plaques, which was also associated to infiltration of inflammatory cells (Stankovic et al. 2019). Also, increased level of IL-33 was associated with thrombotic complications and progression of carotid atherosclerosis in patients with rheumatoid arthritis (Dhillon et al. 2013). Neutrophils are one of the key target cells for IL-33 that exacerbates tissue damage and inflammation by triggering smooth muscle cells death in advanced atherosclerosis (Alves et al. 2010, Silvestre-Roig et al. 2019). Moreover, neutrophil extracellular traps (NETs) expelled from suicidal neutrophils have been detected in atherosclerotic lesion of human and mice and are shown to induce proinflammatory response by inducing the activation of endothelial cells (Megens et al. 2012; Quillard et al. 2015; Warnatsch et al. 2015). Although neutrophils infiltration destabilizes the atherosclerotic plaque (Silvestre-Roig et al. 2019), the interplay between VSMCs, neutrophils and IL-33 in atherosclerosis is not fully understood. Also, little is known about the immunomodulatory effect of CC uptake in VSMCs on atherosclerotic microenvironment. Thus, the aim of the present study was to investigate the cellular and molecular mechanism of CC uptake in VSMCs from healthy donors and to examine the immunomodulatory effect of CC uptake in VSMCs on neutrophil proinflammatory profile including neutrophil extracellular traps (NETs) formation.

## Materials and methods

### Preparation of monohydrate cholesterol crystals (CC)

CC were prepared as described elsewhere (Samstad et al. 2014). Briefly, 100 mg of ultrapure cholesterol (Sigma Aldrich) was dissolved in 50 ml of 1-propanol. The solution was mixed with sterile water (1:1.5) and allowed to rest for 20 min for the crystals to stabilize. All the steps were performed in sterile condition and at room temperature. 1-Propanol was removed by evaporation and CC were resuspended in PBS/0.05% human serum albumin (HSA). CC were further tested for endotoxin contamination using Limulus amebocyte lysate assay (LAL) and LPS levels were found to be below the detection limit (0.01EU/ml). The prepared CC were of size range 1–2 μm and stored at 4 °C.

### Immunohistochemistry

Human carotid atherosclerotic plaques were obtained from patients (n = 3) undergoing carotid endarterectomy at the Division of Thoracic and Cardiovascular Surgery, Örebro University Hospital, Sweden. Informed written consent was obtained from all participants. The use of human atherosclerotic lesions was ethically approved by Uppsala Regional Ethical Board (Dnr 2015/532). The study was ethically performed as per to the guidelines of Helsinki Declaration. The tissues were formalin fixed and paraffin embedded at the Department of Pathology, Örebro University Hospital, Sweden. The paraffin embedded tissues were sectioned (4 µm), dewaxed and rehydrated using Tissue Clear (Sakura, Alphen aan den Rijn, The Netherlands) and decreasing concentration of ethanol. The tissue sections were pretreated with Diva decloaking buffer pH 6 (Biocare Medical, Pacheco, CA, USA) or 10 min at 110 °C to facilitate antigen retrieval. The further staining was performed using primary antibodies against smooth muscle actin (SMA; M0851; Dako; 1:500) and CD68 (NCL‐L‐CD68, Novacastra, Newcastle, UK; 1:50), diluted in Da Vinci Green (Biocare Medical) and incubated for 1 h at room temperature. The slides were then incubated with AP polymer detection kit for visualization of SMA and CD68 using Warp Red. The tissue sections were counterstained with Hematoxylin, dehydrated in ascending grade of ethanol prior to mounting with Pertex mounting medium (Histolab, Gothenburg, Sweden). The slides were scanned using the digital scanner Panoramic 250 Flash III (3DHistech, Budapest, Hungary) and micrographs were analyzed from the Case Viewer using autosettings (Open source version 2.0 software; 3DHistech; https://www.3dhistech.com/).

### Source of data

An existing single cells RNA (scRNA) sequencing dataset from human carotid atherosclerotic plaques from Slenders and coworkers (Slenders et al. 2022) via the PlaqView website (http://plaqviewv2.uvadcos.io/) was used to examine the expression levels of IL-33 and its receptor IL1RL1 and generate Uniform manifold approximation and projection (UMAP) visualization plot of IL-33 and IL1RL1 gene expression in different cell types of carotid plaque.

### Cell cultures

Human umbilical vein endothelial cells (HUVECs) were purchased from Thermo Fisher Scientific (Waltham, MA, USA) and cultured in VascuLife basal medium supplemented with VascuLife VEGF Life factors kit (Lifeline Cell Technology, Frederick, MD, USA) including antibiotics [Penicillin (0.1 U/ml) + Streptomycin (100 ng/ml)] (Thermo Fisher Scientific, Waltham, MA, USA). Human aortic smooth muscle cells (HAoSMCs) from different donors (cat# C-007-5C, Lot# 2164581, 27-year-old, Male; cat# CC-2571, Lot# 0000369150, 43-year-old, Male; cat# CC-2571, Lot# 0000335663, 22-year-old, Male; cat# 354-05a, Lot# 1596, 53-year-old, Male) were purchased from Cell Applications (San Diego, CA), and Lonza (Walkersville, MD). HAoSMCs were cultured using 231 smooth muscle cell culture medium (Gibco, Carlsbad, CA) containing recommended cell growth supplements and antibiotics. Monolayers of all vascular cell were maintained in a humidified incubator with 5% CO2 at 37 °C and used between passage five and nine for all experiments. The culture medium was changed every 72–96 h and subculturing was performed upon confluency. Confluent flasks of vascular cells were, trypsinised using 1X trypsin (Gibco, Life technologies Limited), and seeded to flask or plates for subculturing and experiments, respectively. For experiments, cells were seeded in 6 well (1.5 × 10^5^ cells/well) and 12 well (7 × 10^4^ cells/well) plates maintaining the culturing condition as stated above. CC treatments in HAoSMCs were performed in 231 smooth muscle cell culture media without growth supplements) for 24 h. Treatments with inhibitors were performed 1 h prior to CC treatment. Cell and crystal free supernatants (CC conditioned medium, CC CM) were collected after 24 h of CC treatment and stored at − 20 °C until use. Control conditioned medium (Control CM/CTL CM) are the cell free supernatants from untreated cells. All the inhibitors used in the study are detailed in Additional file 1: Table S1. HAoSMCs are termed as VSMCs in the manuscript. HAoSMCs are referred as VSMCs in the article.

### Isolation of polymorphonuclear neutrophils

Peripheral blood from healthy blood donors was collected in EDTA tubes at Örebro University Hospital. Human neutrophils were prepared from buffy coats obtained at the Örebro University Hospital blood central. According to Swedish law, the use of anonymized buffy coats does not require specific ethical approval. Human neutrophils were isolated from whole blood using Polymorphoprep (Axis-Shield PoC AS, Oslo, Norway) and Lymphoprep (Axis-Shield PoC AS, Oslo, Norway) by density gradient centrifugation, followed by neutrophils collection in the PBS. Erythrocyte were lysed by hypotonic shock and neutrophils were resuspended in the desired medium as per to the treatment. The viability of the neutrophils was > 90% as determined by the trypan blue exclusion test.

### LDH assay

Cell viability was evaluated by Pierce Lactate dehydrogenase (LDH) cytotoxicity assay in the supernatants according to the manufacturer’s instructions. The absorbance was read using the Cytation 3 plate reader (BioTek, Winooski, VT, USA).

### Flow cytometry

HAoSMCs, untreated or treated with CC and inhibitors as described above were harvested and resuspended in FACS buffer (PBS/0.1% FBS/1 mM EDTA). Cells were analyzed on a Gallios flow cyotmeter and data were analyzed using Kaluza software (Beckman Coulter). The gating was set according to shift in side scatter (SSC) from untreated control cells and the quantification of CC uptake is the percentage of cells with high granularity shift indicated by a shift in side scatter (SSC)^high,^ also decribed elsewhere (Donat et al. 2019). Flow cytometry gating strategies are shown in Additional file 2: Figure S2–4. The cells were labelled using 7AAD (Biolegend) to assess the viability. The cells negative for 7AAD staining were considered as live cells. CC uptake in neutrophils was identified by gating for human CD66b [Bv421 PE-Cy7 Mouse Anti-Human CD66b (clone G10F5, Biolegend, Cat#305116)] positive viable cells and the shift in granularity. List of inhibitors used for screening of pathways are displayed in Additional file 1: Table S1.

### Western blotting

Western blotting was used to evaluate the PI3K pathway inhibition in HAoSMCs using wortmannin (10 uM) prior to CC treatment for 6 and 8 h. Total protein extracted from cells using ice cold 1X RIPA buffer (#20188, Millipore) containing protease inhibitor cocktail (#B14001, Bioconnect life sciences) were quantified with BCA protein Assay kit (Thermo Fischer Scientific, Waltham, MA, USA) according to manufacturer’s instruction. Cell lysate were mixed with 4 × SDS sample buffer (SDS, Sigma-Aldrich) and denatured at 95 °C for 5 min. Protein lysates (10–12 μg) were separated using SDS-PAGE (8–16% Criterion™ TGX Stain-free ™ Precast Gels), 10 × Tris/Glycine/SDS running buffer; Biorad, USA) and transferred to nitrocellulose membranes (BioRad, USA) using a Trans-Blot® Electrophoretic Transfer Cell (BioRad, USA) and 10xTris/Glycine transfer buffer (BioRad). Proteins were visualized using a reversible protein stain (Memcode; Thermo Fischer Scientific, Waltham, MA, USA). The membranes were blocked using 5% skim milk/ TBS-T [10 mM Tris–HCl pH 8.0, 150 mM NaCl, 0.1% (v/v) Tween-20] for an hour and further probed for antibodies (detailed in Additional file 1: Table S2) at 4ºC overnight, followed by incubation with horseradish peroxidase (HRP)-conjugated goat anti rabbit or horse anti mouse secondary antibody. Proteins were visualized using Enhanced Chemi Luminescence (ECL) reagent (Western-Ready ECL Substrate premium Kit,BioLegend #426319) and chemiluminescence was detected by ChemiDoc™ MP Imaging system (BioRad,USA). Bands were quantified using Image Lab Software (Bio-Rad, USA).

### Localization of CC in VSMCs using confocal microscopy

VSMCs at density of 10^5^ cells/well were cultured and treated with 0.5 mg/ml of CC in fibronectin-coated (10 ug/ml, 2 h; R and D systems, UK) eight-chamber culture slides for 24 h. The cells were then fixed using ice-cold 4% paraformaldehyde for 30 min at room temperature, followed by incubation with ice-cold PBS containing 0.1% Triton-X100 for 10 min. The cells were washed with ice-cold PBS and blocked in 1% BSA in PBS containing 0.1% Triton-X100 for 30 min. F-actin was visualized by staining with Rhodamine phalloidin, 400X (Invitrogen, Stockholm, Sweden) for 20 min in dark followed by staining the nucleus using 4ʹ,6-Diamidino-2-Phenylindole hydrochloride, (DAPI, Sigma-Aldrich, Germany) for 5 min in dark and washed twice with PBS. Slides were airdried and mounted using antifade reagent and were stored in the dark at 4 °C until viewed under microscope. CC were visualized under polarized light and Images were acquired at 20X and 40X magnification and 1024 × 1024 resolution using confocal laser scanning microscope SP8 (Leica, Germany). The images were further processed with LAS X software (Leica, Wetzlar, Germany).

### Detection of neutrophil extracellular traps (NETs) formation using confocal microscopy

Neutrophils were treated with and without 0.5 mg/ml of CC, phorbol 12-myristate 13-acetate (PMA, 100 nM), and conditioned medium in eight-chamber culture slides for 3 h. Phorbol 12-myristate 13-acetate (PMA) (10 nM/3 h) treatment was used as positive control for NET formation. Cells were washed gently and fixed with 4%paraformaldehyde for 30 min at room temperature, followed by incubation with ice-cold PBS containing 0.1% Triton-X100 for 10 min. The cells were blocked in 1% BSA in PBS containing 0.1% Triton-X100 for 30 min and stained for F actin using Rhodamine phalloidin 400X, for 20 min in the dark. Nucleus was stained using 2.5 uM of Sytox™ green DNA stain for 5 min in dark. Slides were mounted using antifade fluorescence reagent and kept in dark at 4 °C until viewed under microscope.

To quantify NETs, images were processed using ImageJ/FIJI software. The NET-forming population was identified using stringent parameters, including differential Sytox average size, intensity, and shape deviation. Sytox Green images were converted to binary format, and image stacks were generated. These images were then converted to 8-bit grayscale, and a threshold (Triangle threshold) was applied to the stacked images. Particle analysis was performed using a Region of Interest (ROI) manager, with ROIs defined by size (micron^2^): 68 to infinity (in pixel units) and circularity from 0.00 to 1.00. The average size and number of particles (ROIs) were evaluated. NET surface-based threshold of > 68 µm^2^ was used as described elsewhere (van der Linden et al. 2017). Relative Fluorescence Unit (RLU) was determined by calculating the Corrected Total Nucleus Fluorescence (CTNF) of NETs, which is the integrated fluorescence intensity (nuclear area of each cell multiplied by the mean fluorescence of the selected area), using ImageJ/FIJI software.

### Enzyme linked immunosorbent assay

Enzyme Linked immunosorbent Assay was performed to quantify CC dependent IL-33 release in the supernatants of VSMCs using DuoSet® ELISA kit (R&D Systems, Minneapolis,USA) according to manufacturer’s instructions. The absorbance was read at 450 nm using the Cytation 3 plate reader (BioTek, Winooski, VT, USA).

### Neutrophil labelling and neutrophil adhesion assay

Neutrophils were labeled with calcein AM (Molecular Probes, Eugene, OR). In brief, neutrophils were labelled by incubating 5 × 10^6^/ml cells with 50 mg of calcein AM for 30 min at 37 °C in 18 ml of FACs buffer. Cells were then washed twice with PBS at 23 °C and resuspended in the complete VascuLife medium.

Endothelial cells were cultured in 96 well plates and incubated for 48 h with different treatments including conditioned medium (ratio 8:2) and IL-33 positive control. Isolated neutrophils were labelled with calecin AM, 3 ug/ml (#2049068, invitrogen) for 30 min. Endothelial cells were washed and incubated with labelled neutrophils for 30–40 min. Endothelial cells were washed and the fluorescence was measured.

### Olink proteomics

Cell lysates and culture medium from HAoSMCs treated with and without CC were analyzed utilizing Inflammation panels, Cardiovascular III (CVDIII) and Cardiometabolic panels. Additional information about Olink Proseek Multiplex Assay and Gene ontology analysis can be found elsewhere (Paramel et al. 2020).

### Cytokine screening using Bio-Plex 200 system

Human PMA were treated with CC (0.5 mg/ml) and with conditioned medium from CC treated VSMCs respectively for 24 h in 231 smooth muscle cell culture media with supplements. The supernatants were pooled from treatment on PMA from 3 different donors for the cytokine screening using Bio-Plex Pro Human Cytokine 48-Plex Screening Panel. Bio-Plex Manager version 6.1.1 Build 794 were used to analyze the results.

### Phagocytosis assay

Uptake of *E. coli* Bioparticles Conjugated with pHrodo ™ Red and CC were used to quantify phagocytosis in neutrophils using flowcytometry. Cells (1 × 10^5^ cells/100 ul) were incubated in different treatment conditions with pHrodo ™ Red conjugated E. coli Bioparticles and CC for 1 h and 3 h. Cells were analyzed on Gallios flow cytometer and data were analyzed using Kaluza software (Beckman Coulter). Flow cytometry gating strategy is shown in Additional file 2: Figure S3, S4.

### Reactive oxygen species (ROS) measurement

Total ROS production from neutrophils were measured using a luminol-horseradish peroxidase (HRP) assay. The conditioned medium was pre-incubated with control IgG1 or 1 ug/ml of Anti-hIL-33-IgG prior to the addition of 100 ng/ml of recombinant IL-33 for 30 min. The neutrophils (10^6^) were added and incubated with luminol (0.1 mg/ml, Sigma) and HRP (4 U/ml, Roche). Luminescence was measured every third min for 6 h, as previously described (Demirel et al. 2020).

### Statistical analysis

Statistical analysis of the protein multiplex data was done using GraphPad Prism (version 9; GraphPad Software, San Diego, CA, USA; https://www.graphpad.com/scientific-software/prism/). Statistically significant differences between more than two treatment groups were assessed using one-way analysis of variance (ANOVA) with Bonferroni post hoc corrections (for normally distributed data). All results are represented as mean ± SD. For comparison between two treatment groups students t test, Wilcoxson signed rank test and Mann–Whitney were used (for data not normally distributed) and results were represented as mean ± SD or median. *p*-values ≤ 0.05 was considered statistically significant.

### Ethics statement

Informed written consent was obtained from all participants. The use of human atherosclerotic lesions was ethically approved by Uppsala Regional Ethical Board (Dnr 2015/532). The study was ethically performed as per to the guidelines of Helsinki Declaration.

## Results

### VSMCs and macrophages were localized around CC clefts in human carotid plaques

**Fig. 1:**
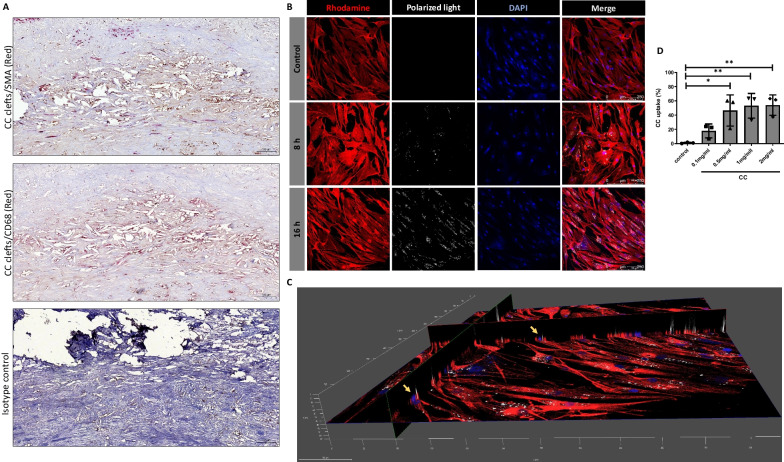
Localization of VSMCs and CC in human atherosclerotic plaque. **A** Immunohistochemistry was performed on paraffin embedded sectioned plaques which were stained for SMA and CD68 positive cells. **B** Confocal microscopy was performed to visualize CC uptake in VSMCs at 8 h and 16 h (CC shown in white by polarized light, Actin in red by Rhodamine phalloidin, and nucleus in blue by DAPI staining), Scale bar, 250 μm. **C** Z stacking 3D image showing CC, nucleus and actin filament (Yellow arrows) in the same plane confirming the uptake. Scale bar, 100 μm, Objective 20X. **D** Dose dependent uptake of CC in VSMCs for 24 h. Data are representative of experiments from VSMCs of 4 donors and displayed as mean ± SD. *p < 0.05, **p < 0.01

CC are known to be found in human atherosclerotic lesions. To further investigate the interaction of CC with vascular cells, we examined the localization of macrophages and smooth muscles in CC rich areas of human atherosclerotic lesions. CC clefts were found in the necrotic core. Sections of human atherosclerotic lesions showed SMA and CD68 positive cells in proximity of “CC clefts” in necrotic core (Fig. 1A). Localization of SMA and CD68 positive cells around the CC clefts indicates the possible interaction of CC to smooth muscle cells and macrophages.

### Phosphoinositide 3-kinase (PI3K) inhibition reduces CC uptake in VSMCs

We next determined CC uptake in primary VSMCs from different donors. Internalization of CC was observed using confocal microscopy. Representative images of VSMCs treated with CC are presented in (Fig. 1B). The Z stacking of the acquired images shows nucleus (DAPI), F actin (Rhodamin phalloidin) and CC (polarized light) in the same plane (Fig. 1C), confirming internalization CC in VSMCs.

A dose-dependent increase in CC uptake by VSMCs was observed using flow cytometry (Fig. 1D). Around 50–60% of VSMCs exhibited saturated cholesterol uptake at a CC concentration of 0.5 mg/ml. A moderate dose dependent increase in the cytotoxicity was observed in response to CC (Additional file 2: Figure S1). CC at a concentration of 0.5 mg/ml was preferred for further experiments.

**Fig. 2:**
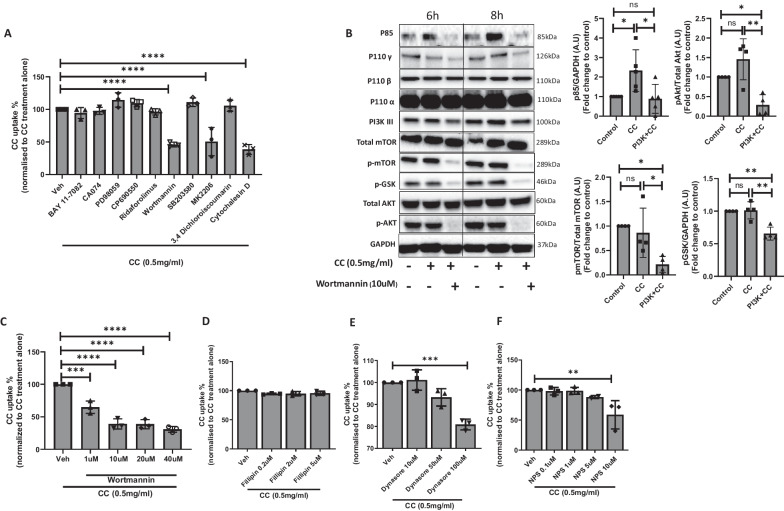
CC uptake mechanism in VSMCs. **A** VSMCs were pre-incubated with DMSO (vehicle, Veh), NF-κB inhibitor BAY 11–7082 (5 μM), cathepsin B inhibitor CA074 (100 μM), ERK1/2 inhibitor PD98059 (10 μM), JAK inhibitor CP690550 (10 μM), AKT inhibitor MK-2206 (1 μM), mTOR inhibitor Ridaforolimus (1 μM), PI3K inhibitor Wortmannin (1 μM), p38 MAPK inhibitor SB203580 (10 μM), serine protease inhibitor 3,4-Dichloroisocoumarin (DCI, 100 μM), and actin inhibitor Cytochalasin D for 1 h prior to CC treatment for 24 h to screen the signaling pathway. **B** Representative western blot bands showing the expression of PI3K and its downstream proteins in response to CC and wortmannin. The densitometry analysis of significantly altered proteins is shown on right side of the blots. Dose dependent response of PI3K inhibitor (wortmannin) (**C**), cholesterol binding protein, filipin (**D**), dynamin GTPase activity inhibitor, dynasore (**E**), and calcium sensing receptor inhibitor, NPS (**F**) for 1 h prior to CC uptake in VSMCs. Data are representative of experiments from VSMCs of 4 donors and displayed as mean ± SD. * *p* < 0.05, ** *p* < 0.01, *** *p* < 0.001

We further investigated the signaling and endocytosis pathways involved in CC uptake in human VSMCs and found that inhibition of PI3K protein and its downstream protein, protein kinase B (AKT) showed significant reduction of CC uptake in VSMCs (Fig. 2A). Inhibition of PI3K signaling using wortmannin significantly reduced P85 of PI3K subunits, however the subunits P110α, P110β, and P110gama remain unaltered (Fig. 2B). Treatment with cytochalasin D and wortmannin reduced CC uptake in VSMCs by inhibition of F-actin and phosphatidylinositol 3′-kinase respectively (Fig. 2A, C). CC uptake was reduced to 50–60% in response to inhibitor Wortmannin and Cytochalasin D, most likely by inhibiting macropinocytosis and phagocytosis pathways. The downstream signaling targets of PI3K pathway, phospho AKT and phospho mTOR were also found reduced upon wortmannin treatment (Fig. 2B). A dose dependent reduction in CC uptake was observed with increasing concentration of wortmannin treatment (Fig. 2C). To further investigate the additional endocytic pathways involved in CC uptake, VSMCs were incubated in the presence of inhibitors of endocytosis pathways, Fillipin III, NPS-2143, and dynasore at a non-cytotoxic concentration. Fillipin III, inhibitor of caveolin-dependent endocytosis showed no effect on CC uptake in human SMC (Fig. 2D). A significant but moderate reduction in CC uptake was observed with 100 µM of dynasore, inhibitors of clathrin-mediated endocytosis in human SMCs (Fig. 2E). NPS-2143, inhibitor of calcium ion sensing receptor mediated constitutive macropinocytosis showed significant reduction in CC uptake at 10 µM concentration (Fig. 2F). Taken together, we show that PI3K facilitates CC uptake in VSMCs by macropinocytosis and phagocytosis.

### VSMCs in response to CC alters inflammatory proteins

**Fig. 3:**
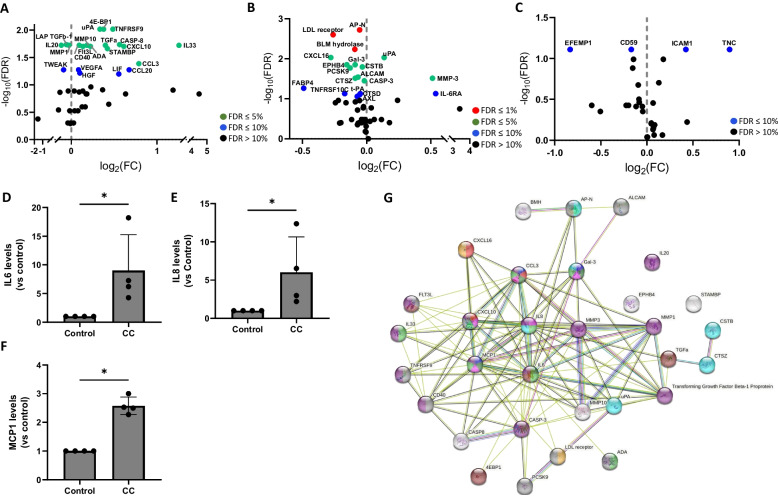
Differential expression of proteins in response to CC in VSMCs (n = 4 donors) using Olink proteomics panels. Volcano plot displaying proteins differentially expressed between control and CC treated in cell culture supernatant using Inflammation panel (**A**) and in the lysate using CVD III (**B**) and Cardiometabolic (**C**) panels. Colors represent FDR levels (red, FDR ≤ 1%; green, FDR ≤ 5%; blue, FDR ≤ 10%; black, FDR > 10%). The labeled dots represent proteins that were differentially expressed in response to CC treatment versus control VSMCs (FDR ≤ 10%). Levels of IL6 (**D**), IL8 (**E**) and MCP-1 (**F**) were measured using ELISA. Data are representative of experiments from VSMCs of 4 donors and displayed as mean ± SD. * p < 0.05. **G** The protein–protein interaction network as analyzed by String software. The red, dark green, pink, and violet nodes represents proteins involved in T cell, monocyte, neutrophil and macrophage chemotaxis, respectively. The green, yellow, blue, purple, grey and brown nodes represents proteins involved in regulation of leukocyte migration, low-density lipoprotein particle receptor activity, neutrophil activation, cytokine signaling in immune system, external side of plasma membrane, PI3K–AkT signaling pathway. The colored lines represent the different possible association between the proteins. A red line indicates the presence of fusion evidence; a green line indicates neighborhood evidence; a blue line indicates co-occurrence evidence; a purple line indicates experimental evidence; a yellow line indicates text-mining evidence; a light blue line indicates database evidence; and a black line indicates co-expression evidence

We next determined the response of VSMCs to CC by analyzing protein secretion in the cell lysate and conditioned medium. VSMCs were cultured with 0.5 mg/ml of CC for 24 h and the conditioned medium and cell lysate exposed to CC were compared to conditioned medium and cell lysate from untreated controls. Cytokine profiles in the secretome were determined using three different Olink multiplex protein panels of 92 proteins (inflammation panel, CVDII panel and cardiometabolic panel). Inflammation panel was used for the analysis of protein secretion in conditioned medium. CVDII panel and cardiometabolic panel were used to analyze the protein in the cell lysate. The protein panels were selected in relevance to CC response in atherosclerosis and the panels were analyzed separately. Of the 92 proteins in each panel, 44, 42, and 63 proteins were undetectable and excluded from inflammation panel, CVDII panel and cardiometabolic panel respectively. Three proteins (IL6, IL8 and MCP1) were excluded from inflammation panel because the values were in the upper limit of detection and were reanalyzed using ELISA. In the inflammation panel, 12 proteins (4E-BP1, uPA, TNFRSF9, MMP10, TGFa, CASP8, IL-33, CXCL10, STAMBP, CD40, Flt3L, ADA) were upregulated, and 3 proteins (LAP TGFb-1, IL20, MMP1) were downregulated in response to CC in VSMCs compared to untreated controls (p-value < 0.05, FDR 5%) (Fig. 3A). CVDII panel showed, 2 upregulated proteins (uPA, MMP-3), and 11 downregulated proteins (AP-N, LAL receptor, BLM hydrolase, CXCL16, Gal-3, EPHB4, CSTB, PCSK9, ALCAM, CTSZ, CASP-3) in the cell lysate of VSMCs in response to CC compared to untreated controls (p-value < 0.05, FDR 5%) (Fig. 3B). None of the protein showed any difference between the CC treated and untreated groups in the cardiometabolic panel (p-value < 0.05, FDR 5%) (Fig. 3C), although 2 proteins (ICAM1, TNC) were found upregulated, and 2 proteins (EFEMP1, CD59) were downregulated when analyzed using FDR 10% (Fig. 3C). Additional file 1: Table S3 shows the list of significantly altered proteins (FDR ≤ 10%) with respective fold changes, p-value and q-value (FDR ≤ 10%). The release of IL6, IL8 and MCP-1 were found significantly upregulated in response to CC in VSMCs from all the 4 donors (Fig. 3D–F).

The significantly altered proteins from the proteomics data was further analyzed using the database STRING to generate interaction network. The gene ontology analysis identified protein–protein interaction network comprising of proteins involved in the regulation of leukocyte migration, low-density lipoprotein particle receptor activity, neutrophil activation, cytokine signaling in immune system, external side of plasma membrane, PI3K–AkT signaling pathway (Fig. 3G).

### Conditioned medium from CC treated VSMCs induce ROS production in neutrophils and NETs formation

**Fig. 4:**
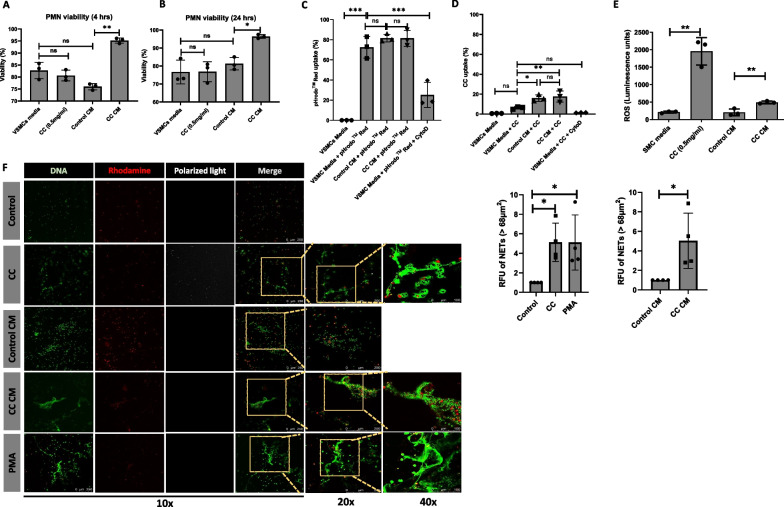
Neutrophil ROS production and NETs formation in response to conditioned medium from CC treated VSMCs. PMN were treated with CC, conditioned medium from unstimulated (Control CM) and CC stimulated (CC CM) VSMCs and. Viability of PMN after 4 h (**A**) and 24 h (**B**) of treatments. Phagocytosis of pHrodoTM Red after 1 h (**C**) and CC after 1 h (**D**) in response to the treatments for 1 h in PMN. ROS was measured in response to the treatments at 90 min (**E**). Data are representative of samples from 3 different experiments and displayed as mean ± SD * p < 0.05, ** p < 0.01, *** p < 0.001. NETs formation in unstimulated PMN, CC treated PMN, conditioned medium (CM) from unstimulated VSMCs, conditioned medium from CC treated VSMCs and PMA for 3 h (**F**). Scale bar 250 μm, objective 10X; Scale bar 250 μm, objective 20X; Scale bar 100 μm, objective 40X

Next, we investigated the impact of CC and condition medium generated from VSMCs on neutrophils. First, we measured the viability of neutrophils in response to CC and conditioned medium at 4 h and 24 h. Viability of neutrophils remain unaltered between CC treatment and untreated control at 4 h and 24 h (Fig. 4A, B). However, the conditioned medium from CC treated VSMCs sustained the viability of neutrophils compared to conditioned medium from untreated control at 4 h and 24 h (Fig. 4A, B).

Since neutrophils are known to respond to particulate substrates either by phagocytic clearance or NETs formation, we further assessed the phagocytic activity by quantifying uptake of pHrodo™ Red and CC at 1 h and 4 h. No significant difference was observed in the phagocytosis of pHrodo™ Red and CC at 1 h and 4 h in neutrophils in response to conditioned medium from CC treated VSMCs when compared to conditioned medium from untreated controls (Fig. 4C, D). However, phagocytosis of CC was significantly induced in response to conditioned medium, although no difference was found between conditioned medium from CC treated VSMCs compared to conditioned medium from untreated controls (Fig. 4C, D). The phagocytosis of pHrodo™ Red *E. Coli* bioparticles was significantly reduced by cytochalasin D (Fig. 4C). We next determined the total ROS production by neutrophils in response to conditioned medium from VSMCs pretreated with and without CC for 90 min. We found that conditioned medium from CC treated VSMCs induced neutrophil ROS production when compared to the conditioned medium from untreated controls (Fig. 4E). No significant differences were observed in neutrophil ROS production between neutrophils cultured in VSMCs medium (control) and conditioned medium from untreated VSMC. Neutrophils in response to CC showed a significant increase in the ROS production (Fig. 4E). Thus, the data suggest that conditioned medium from CC treated VSMCs induces ROS production and sustains survival of neutrophils. However, conditioned medium from CC treated VSMCs has limited influence on the phagocytic property of neutrophils.

Furthermore, we investigated whether conditioned medium from CC treated VSMCs promote NETs formation. We found that conditioned medium from CC treated VSMCs induced NETs formation after 3 h of treatment (Fig. 4F). NET formation was also induced in response to CC alone. Altogether, these data shows that cytokine and growth factors produced by VSMCs in response to CC promote ROS production, neutrophil survival, and NETs formation. Furthermore, the generation of NETs and inability to phagocytose can possibly impart an inflammatory milieu in the atherosclerotic microenvironment.

### IL-33 and conditioned medium from CC treated VSMCs promotes neutrophil adhesion

**Fig. 5:**
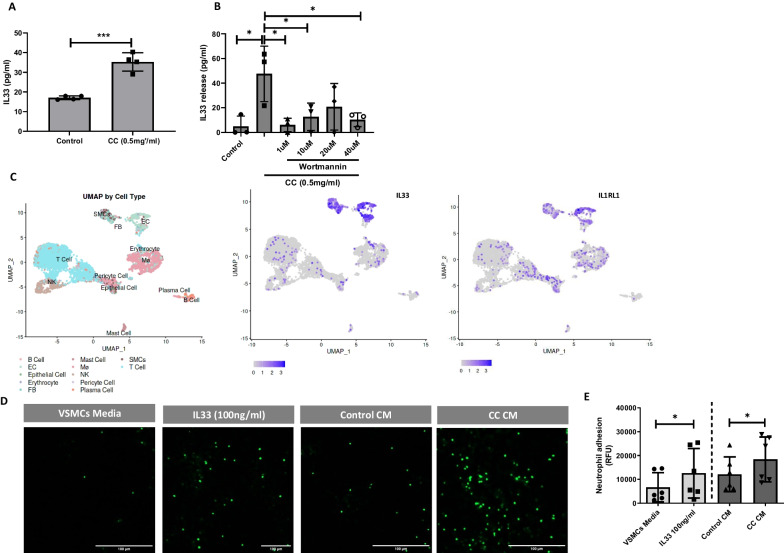
IL33 expression in response to CC in VSMCs. **A** VSMCs were treated with CC for 24 h and IL33 release was measured using ELISA. **B** VSMCs were treated with CC for 24 h that was preincubated with PI3K inhibitor for 1 h. IL33 release was measured using ELISA. **C** Uniform manifold approximation and projection (UMAP) visualization of IL33 and IL1RL1 gene expression in different cell types of carotid plaque from Slenders et al. (n = 38). **D** Representative image showing calecin AB labelled neutrophil adhesion on endothelial cells in response to IL33 alone, conditioned medium (CM) from VSMC treated with and without CC (0.5 mg/ml) for 24 h, Scale bar, 100 μm, objective 4X. **E** Quantification of neutrophil adhesion on endothelial cells in response to treatment. Data are representative of experiments from VSMCs of 4 donors and displayed as mean ± SD.* p < 0.05, *** p < 0.001

Given that IL-33, an alarming cytokine that attracts immune cells in plaque was found upregulated in human atherosclerotic lesions (Stankovic et al. 2019). We further reconfirmed the proteomics showing significant induction of IL-33 release in response to CC in VSMCs using ELISA (Fig. 5A). IL-33 protein showed the highest fold change among the significantly altered proteins in response to CC in VSMCs. The significant increase in the IL-33 release in response to CC was inhibited by the increasing concentration of wortmannin (Fig. 5B), suggesting CC uptake induces IL-33 release in VSMCs which may further promote infiltration of immune cells.

We further explored the expression of IL-33 in human atherosclerotic lesions (n = 38) using single cells RNA (scRNA) sequencing dataset from human carotid atherosclerotic plaques from Slenders and coworkers (Slenders et al. 2022) via the PlaqView website. Both IL-33 and its receptor IL1RL1 were abundantly expressed by SMCs and endothelial cells (Fig. 5C). Moderate levels of IL-33 and IL1RL1 expression were observed in T cells and macrophages (Fig. 5C). We further investigated the effect of IL-33 and conditioned media from VSMCs pretreated with CC on neutrophil adhesion. The endothelial cells were treated with IL-33 alone, conditioned medium from VSMC pretreated with and without CC for 48 h. When neutrophils were incubated for 30 min, IL-33 and conditioned medium from CC treated VSMCs showed a significant increase in neutrophil adhesion compared to untreated controls (Fig. 5D, E).

Thus, these data suggests that IL-33 release upon CC uptake in VSMCs can promote neutrophil adhesion and possibly exacerbate CC-induced inflammatory response in the atherosclerotic microenvironment.

### Neutralization of IL-33 in the conditioned medium reduce NETs formation and neutrophil ROS production

**Fig. 6:**
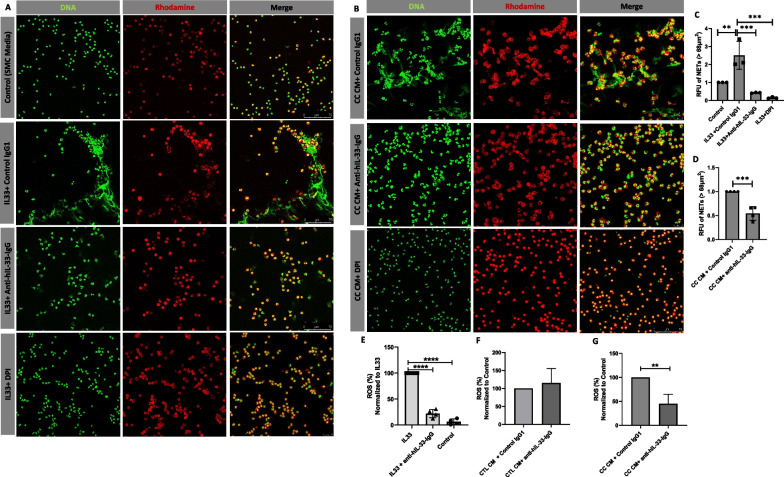
Blocking IL33 in the conditioned medium from VSMCs. **A** PMN were treated with 100 ng/ml of recombinant IL-33 that were pre-incubated with 1ug/ml of Anti-hIL-33-IgG or DPI for 3 h. **B** Conditioned medium from CC treated VSMCs were pre-incubated with 1ug/ml of Anti-hIL-33-IgG or DPI for 3 h. Relative fluorescence intensity of Nets (**C**, **D**). ROS production was measured in PMN in response to 100 ng/ml of recombinant IL-33 (**E**),conditioned medium from unstimulated (Control CM/CTL CM) (**F**) and CC treated (CC CM) VSMCs (**G**) that were pre-incubated with 1ug/ml of Anti-hIL-33-IgG for 30 min. Data are representative of samples from 3 different experiments and displayed as mean ± SD * *p* < 0.05, ** *p* < 0.01, **** *p* < 0.001

Next, we investigated the influence of IL-33 in the conditioned medium from CC treated VSMCs to induce NETs formation and neutrophil ROS production. We show that neutrophil treated with 100 ng/ml of recombinant IL-33 induced NETs formation. Pre-treatment with 1 ug/ml of Anti-hIL-33-IgG or DPI, a ROS inhibitor prior to the treatment with IL-33 showed a reduction in the NETs formation (Fig. 6A). Furthermore, pre-treatment with 1 ug/ml of Anti-hIL-33-IgG or DPI in the conditioned medium from CC treated VSMCs prior to incubation with neutrophils showed a reduction in the NETs formation compared to the conditioned medium from CC treated VSMCs with control IgG1 pre-treatment (Fig. 6B). Nets with size > 68 µm^2^ was not observed in the CC CM + DPI treatment group, suggesting the role of ROS in augmenting the NETs formation.

Furthermore, we also measured the total ROS production in response to recombinant IL-33. IL-33 stimulation significantly induced the ROS production in neutrophils compared to untreated control, which was significantly inhibited by the pre-treatment of 1 ug/ml of Anti-hIL-33-IgG with recombinant IL-33 (Fig. 6E). Pretreatment of conditioned medium from untreated control with Anti-hIL-33-IgG did not alter the ROS production compared to conditioned medium from untreated control with control IgG1 (Fig. 6F). However, Pretreatment of conditioned medium from CC treated VSMCs with Anti-hIL-33-IgG significantly reduced the ROS production compared to the conditioned medium from CC treated VSMCs with control IgG1 (Fig. 6G). This indicates that IL-33 induction by CC in VSMCs play a crucial role in augmenting ROS production and NETs formation in neutrophils, thereby contributing to the inflammatory milieu in human atherosclerotic lesion.

## Discussion

In the present study, we propose a molecular mechanism that integrates VSMCs and neutrophils with CC mediated immune response that is most likely to take place in human atherosclerotic lesions. We show that VSMCs and macrophages are localized around cholesterol clefts suggesting VSMCs might contribute to the CC driven inflammatory response. We also show that VSMCs can phagocytose CC via PI3K pathways and VSMCs upon CC uptake induce the release of inflammatory mediators that promote neutrophils adhesion, ROS production and NETs formation. The present study also demonstrates that IL-33 release upon CC uptake in VSMCs is crucial to promote neutrophil mediated inflammatory response.

Whereas much focus on CC induced response has been attributed towards macrophages, data on CC induced inflammatory response in the VSMCs are scarce. Herein we show that in addition to macrophages, VSMCs were found colocalized with cholesterol clefts in human atherosclerotic lesion. Earlier studies from Ho-Tin-Noé et al. has shown that CC are localized in the atheromatous core, fibrous cap and its interface with the atheromatous core in human plaque (Ho-Tin-Noé et al. 2017). Our observations resemble those of Ho-Tin-Noé et al. who showed localization of CC in the vicinity of SMCs or arising from SMC residues (Ho-Tin-Noé et al. 2017). Using primary cultures of human VSMCs, we further found that VSMCs can phagocytose CC via PI3K–AkT pathway. Although, previous studies have shown independently the phagocytic activity of VSMCs and cholesterol crystal formation in cholesterol loaded VSMCs (Kiyak 1997; Ho-Tin-Noé et al. 2017), this study demonstrate the possibility of VSMCs to uptake CC through phagocytosis in human atherosclerotic lesions. Similar to VSMCs, studies have shown the phagocytic uptake of CC in macrophages, which was abrogated by cytochalasin D (Rajamäki et al. 2010). Moreover, studies have shown that cholesterol crystal induces activation of PI3K, the pharmacological inhibition of which significantly reduced the CC induced IL-1α/β production in macrophages. This fits well with our findings showing that CC activate PI3K signaling pathway, the inhibition of which significantly reduced the CC uptake and CC induced IL-33 production in VSMCs. Hence, these results provide strong support for the hypothesis that CC mediated cellular response largely depends on the PI3K dependent CC uptake in VSMCs. These findings are consistent with Swanson and colleagues, who originally identified PI3K as a key component of phagocytosis and showed that inhibition of PI3K pathway, inhibits macropinocytosis in macrophages (Araki et al. 1996).

It has been shown that VSMCs are the most abundant intimal cells in human fatty streaks and early fibrolipidic lesions. We therefore explored the role of VSMCs in CC-induced inflammation. CC have shown to induce broad spectrum of function in immune response and inflammation (Abela 2010a, b). In the current study we show that several inflammatory mediators such as 4E-BP1, uPA, TNFRSF9, MMP10, TGFa, CASP8, IL-33, CXCL10, STAMBP, CD40, Flt3L, ADA, MMP-3, IL6, IL8 and MCP-1 are markedly induced in response to CC in VSMCs. Several of these proteins play crucial role in atherosclerosis by favoring inflammation, development, and complication of the plaque (Mach et al. 1998; Silence et al. 2001; Falkenberg et al. 2002; Heller et al. 2006; Purroy et al. 2018; Soderstrom et al. 2018; Stankovic et al. 2019). Induction of IL6, IL8 and MCP-1 in response to CC in whole blood have been reported previously (Samstad et al. 2014). In addition, LAP TGFb-1, IL20, MMP1, AP-N, LDL receptor, BLM hydrolase, CXCL16, Gal-3, EPHB4, CSTB, PCSK9, ALCAM, CTSZ, and CASP-3 were found to be downregulated in response to CC in VSMCs. Several lines of evidence suggest that the downregulation of most of these proteins accelerate atherosclerosis. Targeted disruption of CXCL16 and LDL receptor is shown to promote atherosclerosis (Aslanian and Charo 2006). Moreover, studies have shown that release of PCSK9 by VSMCs reduce LDLR levels in macrophages (Ferri et al. 2012), suggesting that our finding on reduced expression of PCSK9 in CC stimulated VSMCs might influence the uptake of cholesterol in the adjacent immune cells at the atherosclerotic lesion site. However, further studies are warranted to explore the influence of reduced levels of PCSK9 in VSMCs in relation to cholesterol crystal uptake. Also, Gal-3 is required for SMCs survival and proliferation (Haigh et al. 2022), the lack of which might influence the survivability of VSMCs. Additionally, deficiency of CASP-3 expression in VSMCs is shown to induce necrosis(Grootaert et al. 2016), thereby suggesting that cholesterol crystal possibly induces necrosis in VSMCs. Although, the combined action of altered proteins has yet to be fully characterized, the physiological action of these altered proteins might vary according to the cell type, type of lesions, and stage of atherosclerosis.

Notably, CC induced IL-33 release was significantly reduced by the inhibition of CC uptake in VSMCs. Elevated expression of IL-33 and its receptor ST2 have been implicated in vulnerable atherosclerotic plaque which was associated with infiltration of inflammatory cells in the plaques. It has been demonstrated that IL-33 is expressed in cholesterol loaded endothelial cells, macrophages, and smooth muscle cells (Stankovic et al. 2019). This is partially consistent with our data that CC uptake in VSMCs induces IL-33 release. IL-33 is an alarming cytokine that belongs to IL1 cytokine family which attract immune cells and elicit proinflammatory response (Cayrol and Girard 2022). Although some studies have indicated atheroprotective role of IL-33 to reduces plaque size (McLaren et al. 2010), other studies have shown IL-33-ST2 levels are increased in patients with carotid atherosclerosis (Stankovic et al. 2019). Our study suggests that CC being an endogenous danger signal induces the expression of IL-33 to attract immune cells like neutrophils to further elevate the immune response which is consistent with studies showing IL-33 promotes infiltration of immune cells (Stankovic et al. 2019). We show that the conditioned medium from CC treated VSMCs and IL-33 promote neutrophil adhesion, suggesting possible role of IL-33 and other inflammatory mediators in the neutrophil infiltration. Also, it was shown that the extent of neutrophil infiltration is associated with pro-inflammatory state and rupture prone lesions (Ionita et al. 2010). This is consistent with our protein interaction data showing that the proteins altered in response to CC are involved in T cell, monocyte, neutrophil and macrophage chemotaxis.

Several lines of evidence suggests that neutrophils are present in the atherosclerotic lesion (Drechsler et al. 2010; Rotzius et al. 2010; Doring et al. 2012). Studies have also shown that neutrophils were localized in the vicinity of CC clefts in human atherosclerotic lesion, thereby providing evidence for the importance of neutrophils in human atherosclerosis (Niyonzima et al. 2020). Furthermore, we show that the viability of neutrophils remains unaltered in response to CC alone when compared to untreated control. However, neutrophils appeared to sustain viability in response to conditioned medium from CC treated VSMCs compared to conditioned medium from untreated control VSMCs, suggesting that the altered expression of several cytokines in the conditioned medium from CC treated VSMCs promoted the viability of neutrophils. We further show that the viability of neutrophils had limited effect on phagocytic function of neutrophils. Although, the conditioned medium from both CC treated VSMCs and untreated control showed increased phagocytosis of CC in neutrophils when compared to CC alone, the conditioned medium from CC treated VSMCs had limited effect on CC phagocytosis when compared to conditioned medium from untreated control. CC by itself in fresh VSMCs media had limited effect on phagocytosis of CC in neutrophils. Unlike CC, increased phagocytosis of pHrodo™ Red was observed in neutrophils in fresh VSMCs media. However, the conditioned medium from CC treated VSMCs had limited effect on pHrodo™ Red phagocytosis when compared to conditioned medium from untreated control. This data on phagocytosis suggests that cytokines produced by VSMCs in response to CC has limited effect on neutrophil phagocytosis but augments neutrophil adhesion and viability. Further studies are needed to explore the conditions required for the CC phagocytosis in isolated neutrophils.

In addition to neutrophil adhesion, we found that conditioned medium from CC treated VSMCs promote neutrophils ROS production and NETs formation. Previous studies have shown that CC are localized in cholesterol rich areas of aortic root in apolipoprotein E (ApoE)–deficient mice and induce NETs formation invitro (Warnatsch et al. 2015). We also show that CC induces NETs formation. Interestingly, we found that IL-33 as the major contributor of neutrophil ROS production and NETs formation. We show that IL-33 neutralization in conditioned medium from CC treated VSMC inhibited neutrophil ROS production and NETs formation. We further show that inhibition of ROS in neutrophil reduced the NETs formation. A recent study has suggested that IL-33 trigger NETs formation by binding to IL-33R on neutrophils and by upregulating CD16 expression. We provide additional mechanism for IL-33 mediated NETs formation via induction of ROS production in neutrophils, which is known to be an important pre-requisite for NET formation (Fuchs et al. 2007; Hakkim et al. 2011; Tembhre et al. 2022). Previous studies have shown that CC trigger NETs formation which further amplifies and drives sterile inflammation in atherosclerotic plaque by priming macrophages for cytokine release, immune cell recruitment and activating T helper 17 cells. Thereby suggesting that employing cellular approach in targeting IL-33 dependent NETosis might help in resolving inflammatory burden of atherosclerotic plaque.

## Conclusions

In conclusion, our data demonstrates a crucial interaction of VSMCs with CC and neutrophils to drive sterile inflammation in atherosclerotic microenvironment. Human atherosclerotic lesion contains VSMCs, CC and neutrophils, with VSMCs and neutrophils known to be in proximity of CC. We found that the crosstalk between VSMCs and CC impart an inflammatory milieu that promotes neutrophil mediated immune response. VSMCs in response to CC promote neutrophil viability but with limited phagocytic clearance of CC by neutrophils. Instead, the increase in IL-33 release in response to CC uptake in VSMCs modulates neutrophils to undergo ROS dependent Netosis which may further attract immune cells and amplifies the inflammation. Future studies are required to determine the impact of employing cellular approach in targeting IL-33 dependent NETosis and to address the systemic effect of such therapeutic interventions.

### Supplementary Information

**Additional file 1: Table S1.** List of inhibitors used for screening signaling pathways. **Table S2.** List of primary and secondary antibodies used for western blot. **Table S3.** Significantly altered proteins detected using Olink multiplex protein panels (inflammation panel, CVDII panel and cardiometabolic panel) from cell lysate and conditioned medium of VSMCs treated with 0.5 mg/ml of CC for 24 h.

**Additional file 2: Figure S1.** Dose dependent cytotoxicity of CC in VSMCs. VSMCs were treated with CC for 24 h. Cytotoxicity of CC on VSMCs was measure by 7AAD staining using flow cytometry. Data are representative of experiments from VSMCs of 4 donors and displayed as mean ± SD. **Figure S2.** Flow cytometry analysis of CCs uptake in VSMCs. Cells were gated on 7AAD negative population according to granularity on side scatter. **Figure S3.** Flow cytometry analysis of pHrodo ™ Red uptake in neutrophils. Cells were gated on pHrodo ™ Red staining according to granularity on side scatter. **Figure S4.** Flow cytometry analysis of CC uptake in neutrophils. Cells were gated on CD66b Bv421 positive staining according to granularity on side scatter.

## Data Availability

The dataset generated and analyzed in this study are available from the corresponding author on reasonable request.

## References

[CR1] Abela GS (2010). Cholesterol crystals piercing the arterial plaque and intima trigger local and systemic inflammation. J Clin Lipidol.

[CR2] Abela GS (2010). Role of cholesterol crystals in myocardial infarction and stroke. Clinical Lipidology.

[CR3] Abela GS, Aziz K, Vedre A, Pathak DR, Talbott JD, DeJong J (2009). Effect of cholesterol crystals on plaques and intima in arteries of patients with acute coronary and cerebrovascular syndromes. Am J Cardiol.

[CR4] Alves JC, Sonego F, Souto FO, Freitas A, Verri WA, Auxiliadora-Martins M, Basile A, McKenzie AN, Xu DM, Cunha FQ, Liew FY (2010). Interleukin-33 attenuates sepsis by enhancing neutrophil influx to the site of infection. Nat Med.

[CR5] Araki N, Johnson MT, Swanson JA (1996). A role for phosphoinositide 3-kinase in the completion of macropinocytosis and phagocytosis by macrophages. J Cell Biol.

[CR6] Aslanian AM, Charo IF (2006). Targeted disruption of the scavenger receptor and chemokine CXCL16 accelerates atherosclerosis. Circulation.

[CR7] Cayrol C, Girard JP (2022). Interleukin-33 (IL-33): a critical review of its biology and the mechanisms involved in its release as a potent extracellular cytokine. Cytokine.

[CR8] Chen Y, Popko B (2018). Cholesterol crystals impede nerve repair. Science.

[CR9] Demirel I, Persson A, Brauner A, Särndahl E, Kruse R, Persson K (2020). Activation of NLRP3 by uropathogenic *Escherichia coli* is associated with IL-1β release and regulation of antimicrobial properties in human neutrophils. Sci Rep.

[CR10] Dhillon OS, Narayan HK, Khan SQ, Kelly D, Quinn PA, Squire IB, Davies JE, Ng LL (2013). Pre-discharge risk stratification in unselected STEMI: is there a role for ST2 or its natural ligand IL-33 when compared with contemporary risk markers?. Int J Cardiol.

[CR11] Donat C, Thanei S, Trendelenburg M (2019). Binding of von Willebrand factor to complement C1q decreases the phagocytosis of cholesterol crystals and subsequent IL-1 secretion in macrophages. Front Immunol.

[CR12] Doring Y, Drechsler M, Wantha S, Kemmerich K, Lievens D, Vijayan S, Gallo RL, Weber C, Soehnlein O (2012). Lack of neutrophil-derived CRAMP reduces atherosclerosis in mice. Circ Res.

[CR13] Drechsler M, Megens RT, van Zandvoort M, Weber C, Soehnlein O (2010). Hyperlipidemia-Triggered Neutrophilia Promotes Early Atherosclerosis. Circulation.

[CR15] Duewell P, Kono H, Rayner KJ, Sirois CM, Vladimer G, Bauernfeind FG, Abela GS, Franchi L, Nuñez G, Schnurr M, Espevik T, Lien E, Fitzgerald KA, Rock KL, Moore KJ, Wright SD, Hornung V, Latz E (2010). NLRP3 inflammasomes are required for atherogenesis and activated by cholesterol crystals. Nature.

[CR16] Falkenberg M, Tom C, DeYoung MB, Wen S, Linnemann R, Dichek DA (2002). Increased expression of urokinase during atherosclerotic lesion development causes arterial constriction and lumen loss, and accelerates lesion growth. Proc Natl Acad Sci U S A.

[CR17] Ferri N, Tibolla G, Pirillo A, Cipollone F, Mezzetti A, Pacia S, Corsini A, Catapano AL (2012). Proprotein convertase subtilisin kexin type 9 (PCSK9) secreted by cultured smooth muscle cells reduces macrophages LDLR levels. Atherosclerosis.

[CR18] Fuchs TA, Abed U, Goosmann C, Hurwitz R, Schulze I, Wahn V, Weinrauch Y, Brinkmann V, Zychlinsky A (2007). Novel cell death program leads to neutrophil extracellular traps. J Cell Biol.

[CR19] Grootaert MO, Schrijvers DM, Hermans M, Van Hoof VO, De Meyer GR, Martinet W (2016). Caspase-3 Deletion Promotes Necrosis in Atherosclerotic Plaques of ApoE Knockout Mice. Oxid Med Cell Longev.

[CR20] Haigh S, Li X, Bordan Z, Sellers H, Meadows ML, Barman S, Fulton D (2022). GAL3 excretion regulates smooth muscle cell survival and proliferation. FASEB J.

[CR21] Hakkim A, Fuchs TA, Martinez NE, Hess S, Prinz H, Zychlinsky A, Waldmann H (2011). Activation of the Raf-MEK-ERK pathway is required for neutrophil extracellular trap formation. Nat Chem Biol.

[CR22] Heller EA, Liu E, Tager AM, Yuan Q, Lin AY, Ahluwalia N, Jones K, Koehn SL, Lok VM, Aikawa E, Moore KJ, Luster AD, Gerszten RE (2006). Chemokine CXCL10 promotes atherogenesis by modulating the local balance of effector and regulatory T cells. Circulation.

[CR23] Ho-Tin-Noé B, Vo S, Bayles R, Ferrière S, Ladjal H, Toumi S, Deschildre C, Ollivier V, Michel J-B (2017). Cholesterol crystallization in human atherosclerosis is triggered in smooth muscle cells during the transition from fatty streak to fibroatheroma. J Pathol.

[CR24] Ionita MG, van den Borne P, Catanzariti LM, Moll FL, de Vries JPPM, Pasterkamp G, Vink A, de Kleijn DPV (2010). High neutrophil numbers in human carotid atherosclerotic plaques are associated with characteristics of rupture-prone lesions. Arterioscler Thromb Vasc Biol.

[CR25] Kiyak JH (1997). Cholesterol crystals, smooth muscle cells and new data on the genesis of atherosclerosis. Pol J Pathol.

[CR26] Li X, Bayliss G, Zhuang S (2017). Cholesterol Crystal embolism and chronic kidney disease. Int J Mol Sci.

[CR27] Libby P (2002). Inflammation in atherosclerosis. Nature.

[CR28] Liew FY, Girard J-P, Turnquist HR (2016). Interleukin-33 in health and disease. Nat Rev Immunol.

[CR29] Lim RS, Suhalim JL, Miyazaki-Anzai S, Miyazaki M, Levi M, Potma EO, Tromberg BJ (2011). Identification of cholesterol crystals in plaques of atherosclerotic mice using hyperspectral CARS imaging. J Lipid Res.

[CR30] Lopez-Castejon G, Brough D (2011). Understanding the mechanism of IL-1 beta secretion. Cytokine Growth Factor Rev.

[CR31] Mach F, Schönbeck U, Sukhova GK, Atkinson E, Libby P (1998). Reduction of atherosclerosis in mice by inhibition of CD40 signalling. Nature.

[CR32] Mani AM, Chattopadhyay R, Singh NK, Rao GN (2018). Cholesterol crystals increase vascular permeability by inactivating SHP2 and disrupting adherens junctions. Free Radic Biol Med.

[CR33] McLaren JE, Michael DR, Salter RC, Ashlin TG, Calder CJ, Miller AM, Liew FY, Ramji DP (2010). IL-33 reduces macrophage foam cell formation. J Immunol.

[CR34] Megens RTA, Vijayan S, Lievens D, Döring Y, van Zandvoort MAMJ, Grommes J, Weber C, Soehnlein O (2012). Presence of luminal neutrophil extracellular traps in atherosclerosis. Thromb Haemost.

[CR35] Nidorf SM, Fiolet A, Abela GS (2020). Viewing atherosclerosis through a crystal lens: How the evolving structure of cholesterol crystals in atherosclerotic plaque alters its stability. J Clin Lipidol.

[CR36] Niyonzima N, Bakke SS, Gregersen I, Holm S, Sandanger O, Orrem HL, Sporsheim B, Ryan L, Kong XY, Dahl TB, Skjelland M, Sorensen KK, Rokstad AM, Yndestad A, Latz E, Gullestad L, Andersen GO, Damas JK, Aukrust P, Mollnes TE, Halvorsen B, Espevik T (2020). Cholesterol crystals use complement to increase NLRP3 signaling pathways in coronary and carotid atherosclerosis. EBioMedicine.

[CR37] Paramel GV, Karadimou G, Eremo AG, Ljungberg LU, Hedin U, Olofsson PS, Folkersen L, Paulsson-Berne G, Sirsjö A, Fransén K (2020). Expression of CARD8 in human atherosclerosis and its regulation of inflammatory proteins in human endothelial cells. Sci Rep.

[CR38] Pervaiz MH, Durga S, Janoudi A, Berger K, Abela GS (2018). PET/CTA detection of muscle inflammation related to cholesterol crystal emboli without arterial obstruction. J Nucl Cardiol.

[CR39] Pichavaram P, Mani AM, Singh NK, Rao GN (2019). Cholesterol crystals promote endothelial cell and monocyte interactions via H(2)O(2)-mediated PP2A inhibition, NFκB activation and ICAM1 and VCAM1 expression. Redox Biol.

[CR40] Purroy A, Roncal C, Orbe J, Meilhac O, Belzunce M, Zalba G, Villa-Bellosta R, Andres V, Parks WC, Paramo JA, Rodriguez JA (2018). Matrix metalloproteinase-10 deficiency delays atherosclerosis progression and plaque calcification. Atherosclerosis.

[CR41] Quillard T, Araújo HA, Franck G, Shvartz E, Sukhova G, Libby P (2015). TLR2 and neutrophils potentiate endothelial stress, apoptosis and detachment: implications for superficial erosion. Eur Heart J.

[CR42] Rajamäki K, Lappalainen J, Öörni K, Välimäki E, Matikainen S, Kovanen PT, Eklund KK (2010). Cholesterol crystals activate the NLRP3 inflammasome in human macrophages: a novel link between cholesterol metabolism and inflammation. PLoS ONE.

[CR43] Rotzius P, Thams S, Soehnlein O, Kenne E, Tseng CN, Björkström NK, Malmberg KJ, Lindbom L, Eriksson EE (2010). Distinct infiltration of neutrophils in lesion shoulders in ApoE-/- mice. Am J Pathol.

[CR44] Samstad EO, Niyonzima N, Nymo S, Aune MH, Ryan L, Bakke SS, Lappegård KT, Brekke OL, Lambris JD, Damås JK, Latz E, Mollnes TE, Espevik T (2014). Cholesterol crystals induce complement-dependent inflammasome activation and cytokine release. J Immunol.

[CR45] Scolari F, Tardanico R, Zani R, Pola A, Viola BF, Movilli E, Maiorca R (2000). Cholesterol crystal embolism: a recognizable cause of renal disease. Am J Kidney Dis.

[CR46] Sedaghat A, Grundy SM (1980). Cholesterol crystals and the formation of cholesterol gallstones. N Engl J Med.

[CR47] Silence J, Lupu F, Collen D, Lijnen HR (2001). Persistence of atherosclerotic plaque but reduced aneurysm formation in mice with stromelysin-1 (MMP-3) gene inactivation. Arterioscler Thromb Vasc Biol.

[CR48] Silvestre-Roig C, Braster Q, Wichapong K, Lee EY, Teulon JM, Berrebeh N, Winter J, Adrover JM, Santos GS, Froese A, Lemnitzer P, Ortega-Gomez A, Chevre R, Marschner J, Schumski A, Winter C, Perez-Olivares L, Pan C, Paulin N, Schoufour T, Hartwig H, Gonzalez-Ramos S, Kamp F, Megens RTA, Mowen KA, Gunzer M, Maegdefessel L, Hackeng T, Lutgens E, Daemen M, von Blume J, Anders HJ, Nikolaev VO, Pellequer JL, Weber C, Hidalgo A, Nicolaes GAF, Wong GCL, Soehnlein O (2019). Externalized histone H4 orchestrates chronic inflammation by inducing lytic cell death. Nature.

[CR49] Slenders L, Landsmeer LPL, Cui K, Depuydt MAC, Verwer M, Mekke J, Timmerman N, van den Dungen NAM, Kuiper J, de Winther MPJ, Prange KHM, Ma WF, Miller CL, Aherrahrou R, Civelek M, de Borst GJ, de Kleijn DPV, Asselbergs FW, den Ruijter HM, Boltjes A, Pasterkamp G, van der Laan SW, Mokry M (2022). Intersecting single-cell transcriptomics and genome-wide association studies identifies crucial cell populations and candidate genes for atherosclerosis. Eur Heart J Open.

[CR50] Soderstrom LA, Tarnawski L, Olofsson PS (2018). CD137: A checkpoint regulator involved in atherosclerosis. Atherosclerosis.

[CR51] Stankovic M, Ljujic B, Babic S, Maravic-Stojkovic V, Mitrovic S, Arsenijevic N, Radak D, Pejnovic N, Lukic ML (2019). IL-33/IL-33R in various types of carotid artery atherosclerotic lesions. Cytokine.

[CR52] Sun Y, Pavey H, Wilkinson I, Fisk M (2021). Role of the IL-33/ST2 axis in cardiovascular disease: a systematic review and meta-analysis. PLoS ONE.

[CR53] Tembhre MK, Sriwastva MK, Hote MP, Srivastava S, Solanki P, Imran S, Lakshmy R, Sharma A, Jaiswal K, Upadhyay AD (2022). Interleukin-33 induces neutrophil extracellular trap (NET) formation and macrophage necroptosis via enhancing oxidative stress and secretion of proatherogenic factors in advanced atherosclerosis. Antioxidants.

[CR54] van der Linden M, Westerlaken GHA, van der Vlist M, van Montfrans J, Meyaard L (2017). Differential signalling and kinetics of neutrophil extracellular trap release revealed by quantitative live imaging. Sci Rep.

[CR55] Varghese GP, Folkersen L, Strawbridge RJ, Halvorsen B, Yndestad A, Ranheim T, Krohg-Sørensen K, Skjelland M, Espevik T, Aukrust P, Lengquist M, Hedin U, Jansson JH, Fransén K, Hansson GK, Eriksson P, Sirsjö A (2016). NLRP3 inflammasome expression and activation in human atherosclerosis. J Am Heart Assoc.

[CR56] Warnatsch A, Ioannou M, Wang Q, Papayannopoulos V (2015). Neutrophil extracellular traps license macrophages for cytokine production in atherosclerosis. Science.

