# Supplementary material for: Vascular smooth muscle cells in response to cholesterol crystals modulates inflammatory cytokines release and promotes neutrophil extracellular trap formation
Source: Mol Med. 2024 Mar 22;30:42. doi: 10.1186/s10020-024-00809-8 (PMC10960408; doi:10.1186/s10020-024-00809-8)
Supplement: Supplementary file 1 — Additional file 1: Table S1. List of inhibitors used for screening signaling pathways. Table S2. List of primary and secondary antibodies used for western blot. Table S3. Significantly altered proteins detected using Olink multiplex protein panels (inflammation panel, CVDII panel and cardiometabolic panel) from cell lysate and conditioned medium of VSMCs treated with 0.5 mg/ml of CC for 24 h. [file 10020_2024_809_MOESM1_ESM.docx]

**Table S1:** List of inhibitors used for screening signaling pathways.

| **Inhibitors** | **Signaling Pathway** | **Manufacturer** | **Concentration (μM)** |
| --- | --- | --- | --- |
| BAY 11-7082 | NFkB | Enzo Life Sciences | 5 |
| Wortmannin | PI3K | Selleckchem | 1 |
| CA074 | Cathepsin B | Apexbio Technology LLc | 100 |
| SB203580 | P38 MAPK | Santa Cruz Biotechnology | 10 |
| MK-2206 | AKT | Selleckchem | 1 |
| Ridaforolimus | MTOR | Selleckchem | 1 |
| PD98059 | ERK1/2 | Santa Cruz Biotechnology | 10 |
| 3,4 Dichloroisocvoumarin | Serine protease | Merck millipore | 100 |
| Cytochalasein D | Actin polymerization | Sigma Aldrich | 2 |

**Table S2:** List of primary and secondary antibodies used for western blot.

| **Primary antibody** | **Primary antibody manufacturer** | **Primary antibody dilution** | **Secondary antibody** | **Secondary antibody dilution** | **Secondary antibody manufacturer** |
| --- | --- | --- | --- | --- | --- |
| p85α | #1637, Santa Cruz | 1 : 200 | Anti Mouse | 1 : 2000 | #7076, Cell Signalling Technology |
| PI3Kinase P110g | #5405T, Cell Signalling Technology | 1 : 1000 | Anti Rabbit | 1 : 2000 | #7074, Cell Signalling Technology |
| PI3Kinase P110b | #3011T, Cell Signalling Technology | 1 : 1000 | Anti Rabbit | 1 : 2000 | #7074, Cell Signalling Technology |
| PI3Kinase P110a | #4249T, Cell Signalling Technology | 1 : 1000 | Anti Rabbit | 1 : 2000 | #7074, Cell Signalling Technology |
| MTOR | #7C1D, Cell Signalling Technology | 1 : 1000 | Anti Rabbit | 1 : 2000 | #7074, Cell Signalling Technology |
| pMToR(ser2448) | #55365, Cell Signalling Technology | 1 : 1000 | Anti Rabbit | 1 : 2000 | #7074, Cell Signalling Technology |
| AKT | #4691, Cell Signalling Technology | 1 : 1000 | Anti Rabbit | 1 : 2000 | #7074, Cell Signalling Technology |
| PI3Kinase class III | #3358, Cell Signalling Technology | 1 : 1000 | Anti Rabbit | 1 : 2000 | #7074, Cell Signalling Technology |
| pGSK3b | #5558T, Cell signaling Technology | 1 : 1000 | Anti Rabbit | 1 : 2000 | #7074, Cell Signalling Technology |
| pAKT(S473) | XP(R), #4060T, Cell Signalling Technology | 1:1000 | Anti Rabbit | 1: 2000 | #7074, Cell Signalling Technology |
| GAPDH | #47724, Santa Cruz | 1 : 5000 | Anti Mouse | 1 : 2000 | #7076, Cell Signalling Technology |

**Table S3.** Significantly altered proteins detected using Olink multiplex protein panels (inflammation panel, CVDII panel and cardiometabolic panel) from cell lysate and conditioned medium of VSMCs treated with 0.5mg/ml of CC for 24 h. Fold change, p-value and q-value (FDR≤10%) are shown.

| **Cardiometabolic Panel** | | | |
| --- | --- | --- | --- |
| **Protein ID** | **Fold Change(log_2_)** | **p-value** | **q-value** |
| EFEMP1 | -0,833156036 | 0,0092 | 0,0777 |
| ICAM1 | 0,423309237 | 0,0095 | 0,0777 |
| CD59 | -0,16909569 | 0,0109 | 0,0777 |
| TNC | 0,896853073 | 0,0141 | 0,0777 |
|  | | | |
| **CVDIII Panel** | | | |
| **Protein ID** | **Fold Change(log_2_)** | **p-value** | **q-value** |
| AP-N | -0,05 | <0,0001 | 0,0019 |
| LDL receptor | -0,26 | 0,0002 | 0,0025 |
| BLM hydrolase | -0,09 | 0,0006 | 0,0058 |
| uPA | 0,14 | 0,0013 | 0,0093 |
| CXCL16 | -0,28 | 0,0015 | 0,0093 |
| Gal-3 | -0,09 | 0,0030 | 0,0141 |
| EPHB4 | -0,15 | 0,0032 | 0,0141 |
| CSTB | -0,03 | 0,0042 | 0,0160 |
| PCSK9 | -0,13 | 0,0049 | 0,0169 |
| ALCAM | -0,07 | 0,0093 | 0,0288 |
| CTSZ | -0,09 | 0,0112 | 0,0306 |
| MMP-3 | 0,51 | 0,0119 | 0,0306 |
| CASP-3 | -0,02 | 0,0150 | 0,0355 |
| FABP4 | -0,49 | 0,0247 | 0,0544 |
| IL-6RA | 0,54 | 0,0364 | 0,0744 |
| t-PA | -0,05 | 0,0398 | 0,0744 |
| TNFRSF10C | -0,17 | 0,0411 | 0,0744 |
| CTSD | -0,05 | 0,0460 | 0,0787 |
| AXL | -0,07 | 0,0539 | 0,0874 |
|  | | | |
| **Inflammation Panel** | | | |
| **Protein ID** | **Fold Change(log_2_)** | **p-value** | **q-value** |
| TNFRSF9 | 0,49 | 0,0004 | 0,0081 |
| 4E-BP1 | 0,38 | 0,0009 | 0,0081 |
| uPA | 0,33 | 0,0011 | 0,0081 |
| CASP-8 | 0,56 | 0,0027 | 0,0154 |
| LAP TGF-beta-1 | -0,06 | 0,0033 | 0,0154 |
| IL33 | 1,26 | 0,0041 | 0,0159 |
| ADA | 0,22 | 0,0054 | 0,0167 |
| Flt3L | 0,14 | 0,0061 | 0,0167 |
| CXCL10 | 0,61 | 0,0065 | 0,0167 |
| STAMBP | 0,42 | 0,0079 | 0,0183 |
| CCL3 | 0,79 | 0,0164 | 0,0345 |
| VEGFA | 0,08 | 0,0250 | 0,0447 |
| CCL20 | 0,68 | 0,0254 | 0,0447 |
| TWEAK | -0,09 | 0,0271 | 0,0447 |
| HGF | 0,10 | 0,0332 | 0,0511 |
| LIF | 0,55 | 0,0368 | 0,0531 |
| MCP-3 | 0,23 | 0,0675 | 0,0917 |
