# Supplementary material for: Vascular smooth muscle cells in response to cholesterol crystals modulates inflammatory cytokines release and promotes neutrophil extracellular trap formation
Source: Mol Med. 2024 Mar 22;30:42. doi: 10.1186/s10020-024-00809-8 (PMC10960408; doi:10.1186/s10020-024-00809-8)
Supplement: Supplementary file 2 — Additional file 2: Figure S1. Dose dependent cytotoxicity of CC in VSMCs. VSMCs were treated with CC for 24 h. Cytotoxicity of CC on VSMCs was measure by 7AAD staining using flow cytometry. Data are representative of experiments from VSMCs of 4 donors and displayed as mean ± SD. Figure S2. Flow cytometry analysis of CCs uptake in VSMCs. Cells were gated on 7AAD negative population according to granularity on side scatter. Figure S3. Flow cytometry analysis of pHrodo ™ Red uptake in neutrophils. Cells were gated on pHrodo ™ Red staining according to granularity on side scatter. Figure S4. Flow cytometry analysis of CC uptake in neutrophils. Cells were gated on CD66b Bv421 positive staining according to granularity on side scatter. [file 10020_2024_809_MOESM2_ESM.pptx]

## Slide 1
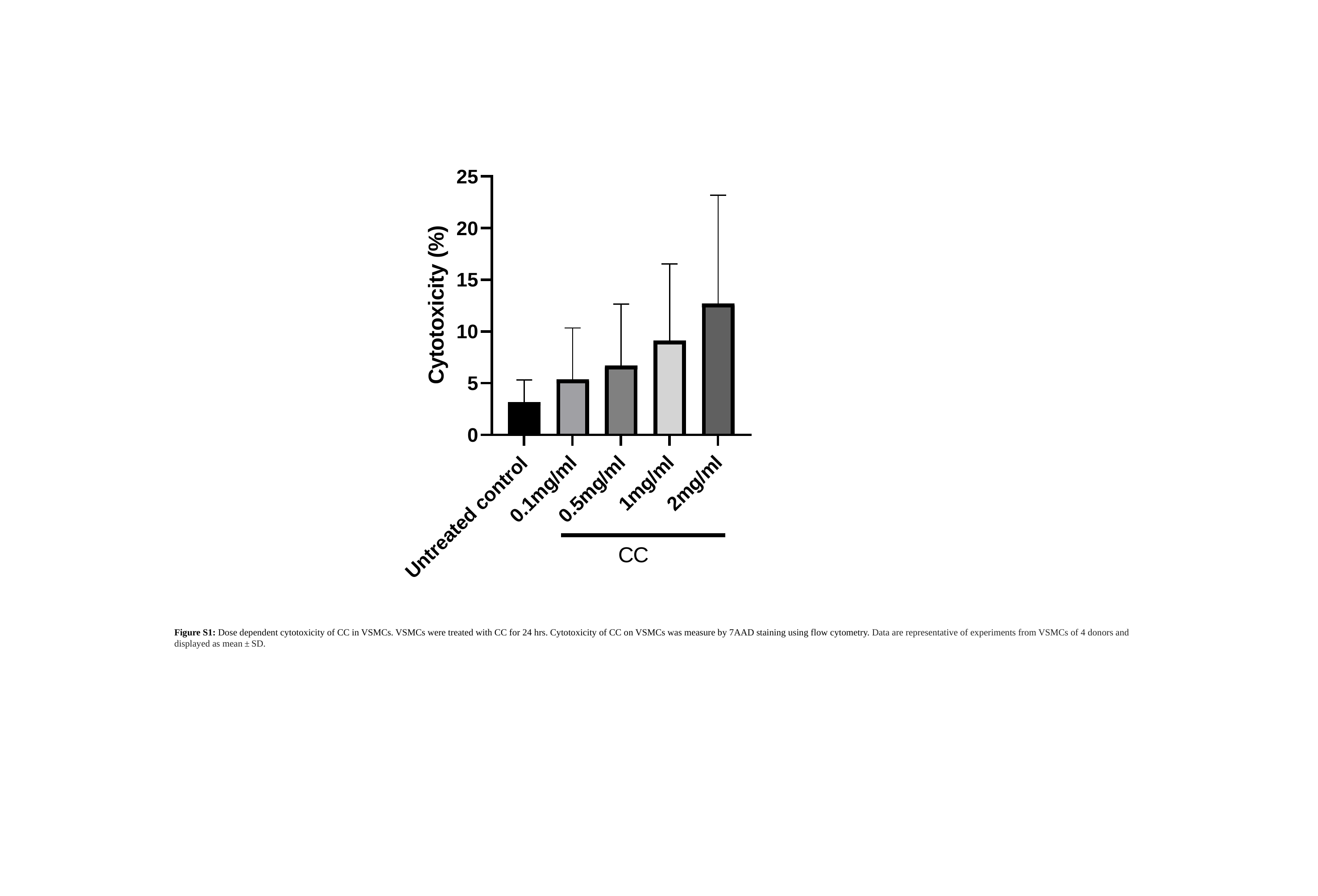

Figure S1: Dose dependent cytotoxicity of CC in VSMCs. VSMCs were treated with CC for 24 hrs. Cytotoxicity of CC on VSMCs was measure by 7AAD staining using flow cytometry. Data are representative of experiments from VSMCs of 4 donors and displayed as mean ± SD.

## Slide 2
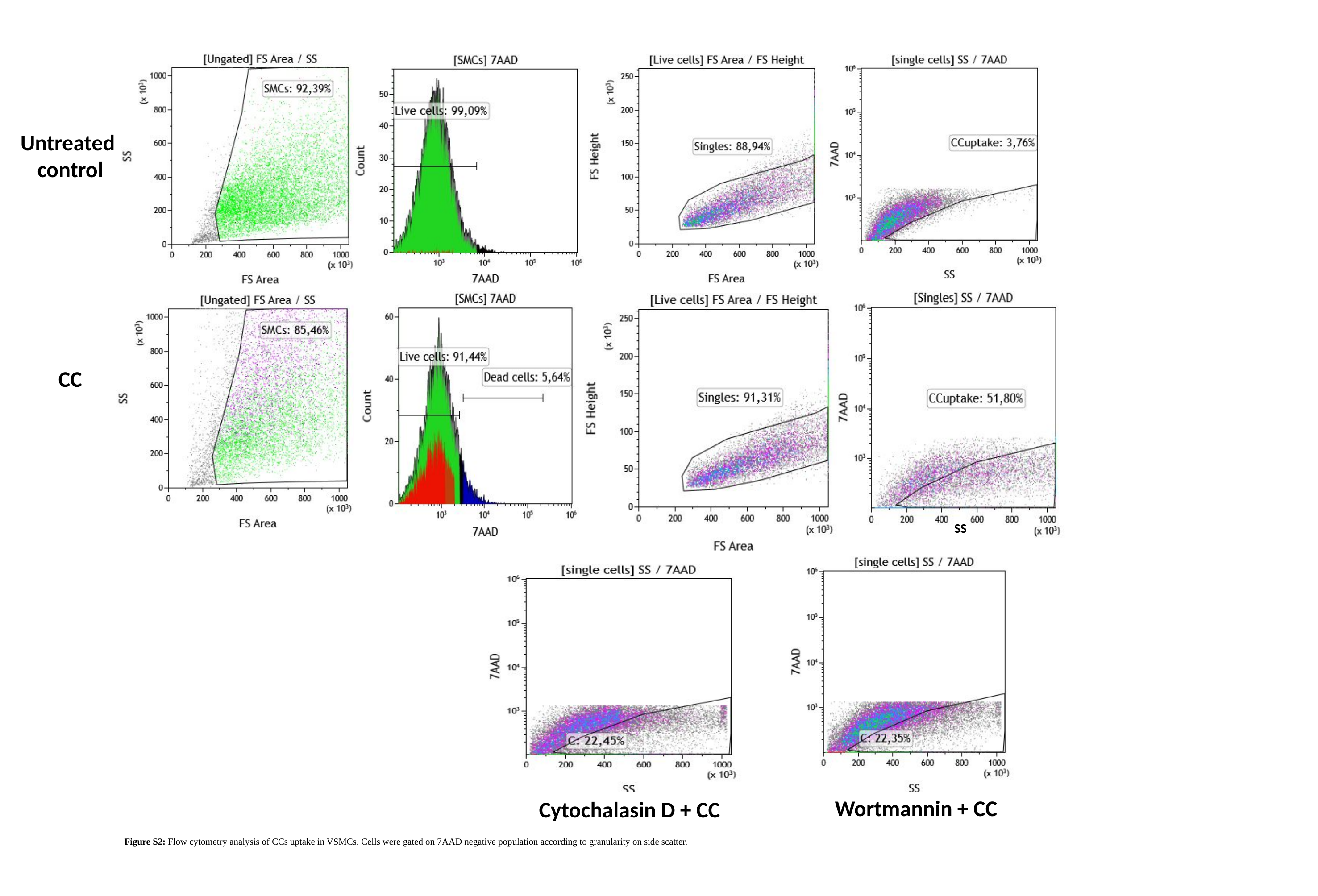

Untreated
control
CC
SS
Wortmannin + CC
Cytochalasin D + CC
Figure S2: Flow cytometry analysis of CCs uptake in VSMCs. Cells were gated on 7AAD negative population according to granularity on side scatter.

## Slide 3
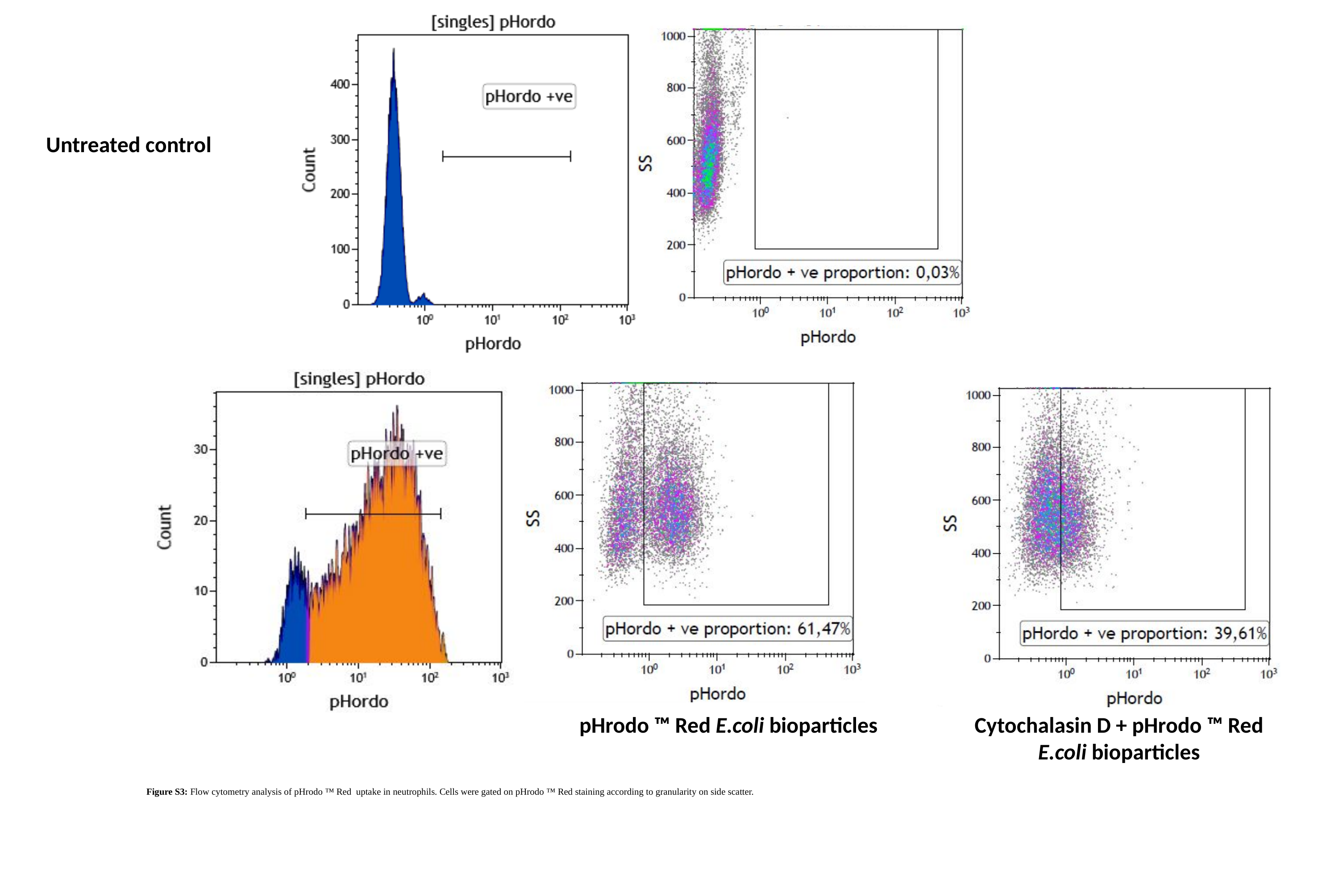

Untreated control
pHrodo ™ Red E.coli bioparticles
Cytochalasin D + pHrodo ™ Red E.coli bioparticles
Figure S3: Flow cytometry analysis of pHrodo ™ Red uptake in neutrophils. Cells were gated on pHrodo ™ Red staining according to granularity on side scatter.

## Slide 4
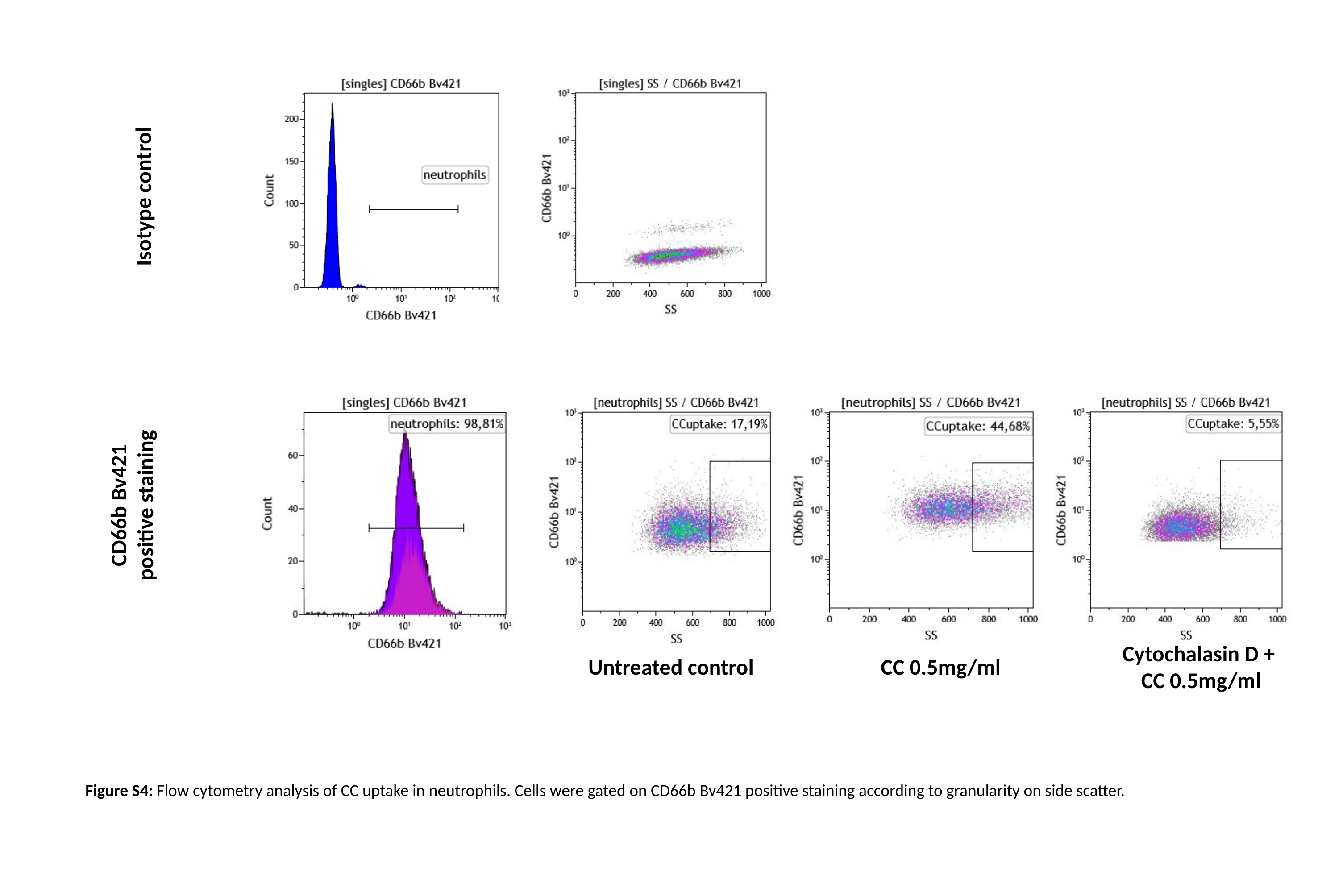

Isotype control
CD66b Bv421 positive staining
Cytochalasin D +
CC 0.5mg/ml
Untreated control
CC 0.5mg/ml
Figure S4: Flow cytometry analysis of CC uptake in neutrophils. Cells were gated on CD66b Bv421 positive staining according to granularity on side scatter.
